# Real-world effectiveness of MiSight 1 Day contact lenses in Taiwanese children: a multicenter retrospective study

**DOI:** 10.3389/fmed.2026.1737902

**Published:** 2026-03-13

**Authors:** Yu-Te Huang, I-Ming Wang, Yu-Hsuan Huang, Pi-Jung Lin, Hao-Lin Su, Tzu-Hsun Tsai, Yao-Lin Liu, I-Jong Wang, Hui-Ju Lin

**Affiliations:** 1 Graduate Institute of Biomedical Sciences, China Medical University, Taichung, Taiwan; 2 Eye Center, China Medical University Hospital, Taichung, Taiwan; 3 Department of Medical Research, China Medical University, Taichung, Taiwan; 4 Shilin Universal Eye Center, Taipei, Taiwan; 5 Universal Eye Center, Taichung, Taiwan; 6 Department of Ophthalmology, National Taiwan University Hospital, Taipei, Taiwan

**Keywords:** axial length, MiSight 1 Day, myopia control, percentile-matched norms, real-world study

## Abstract

**Purpose:**

To evaluate the real-world effectiveness of MiSight 1 Day soft contact lenses for myopia control in Taiwanese children using age-, sex-, and percentile-matched axial length (AL) references.

**Methods:**

Retrospective multicenter study across 23 Universal Eye Center clinics. Children aged 6–18 years who initiated MiSight between October 2020 and July 2022 and completed ≥12 months of follow-up were included. Spherical equivalent (SE) and AL were extracted from clinical records and annualized. Observed AL change was compared with matched Taiwanese normative percentiles to estimate axial suppression. Subgroups included age, baseline AL percentile, and prior treatment history.

**Results:**

A total of 368 eyes from 368 patients were analyzed (mean age 11.8 ± 2.3 years; 52% female; mean follow-up 19.2 ± 5.0 months). Mean SE progression was −0.41 ± 0.42 D/year. Mean AL elongation was 0.17 mm/year, corresponding to a mean AL suppression of 0.06 mm/year relative to matched Taiwanese norms. Visual performance was favorable, with 98% achieving ≥20/25. Outcomes were similar across prior treatment groups, including previous atropine or orthokeratology exposure.

**Conclusion:**

In routine Taiwanese practice, MiSight 1 Day provided meaningful myopia control and maintained good vision. AL growth was reduced by 27% versus matched normative expectations. These findings support the translational effectiveness of MiSight and emphasize the value of regional normative benchmarks for clinical interpretation.

## Introduction

Myopia is a leading cause of visual disability worldwide, with the highest burden in East Asia (1). In Taiwan, recent surveys report myopia in more than 80% of high school students, and about one third meet criteria for high myopia (≤ − 6.00 D). These children face increased risks of myopic maculopathy, retinal detachment, and glaucoma (2). The public health need for effective and scalable myopia control in this population is therefore urgent.

Dual-focus soft contact lenses are a key option among current interventions. MiSight® 1 Day (CooperVision) employs concentric treatment zones that create peripheral myopic defocus while maintaining clear central vision. Randomized clinical trials have shown favorable outcomes. In the 3-year trial by Chamberlain et al. (3), MiSight wearers had 52% less axial elongation and 59% less refractive progression than single-vision spectacle wearers (4). These data support the biological plausibility and clinical utility of the design.

Trial results may not fully represent performance in routine practice. Adherence, refraction methods, and optical quality can vary across clinics and patients. Ethnic differences in ocular growth further complicate generalization. Taiwanese children exhibit faster axial elongation than many European cohorts, which underscores the need for local biometric references when interpreting treatment effects (5). Using Taiwanese normative axial length percentiles offers a population-adapted way to interpret treatment effects. Because axial growth patterns differ across regions, using non-local growth references may lead to misleading conclusions about the magnitude of axial slowing in Taiwanese children. A percentile-based approach compares each child’s observed axial growth with the expected growth for peers of the same age and sex at a similar baseline axial length percentile. This provides a clinically intuitive interpretation of treatment response in the context of the ocular growth trajectory most relevant to Taiwanese practice.

Real-world studies of MiSight are increasing, but few have anchored outcomes to age- and sex-matched axial length percentiles from local populations (4). The influence of prior therapies, including low-dose atropine or orthokeratology, on subsequent MiSight effectiveness also remains insufficiently characterized in Asian cohorts (6).

The aim of this study was to evaluate the real-world effectiveness of MiSight 1 Day in Taiwanese children using multicenter retrospective data. We quantify annual changes in spherical equivalent (SE) and axial length (AL) and benchmark these against Taiwanese age- and sex-specific AL percentiles. We also examine patient-level factors, including prior treatments, that may modify response, to provide population-adapted evidence for clinical practice in Taiwan.

## Methods

### Study design

This was a retrospective, multicenter, observational study across 23 branches of the Universal Eye Center (UEC) network in Taiwan. Clinical records from October 2020 to July 2022 were reviewed. The study adhered to the Declaration of Helsinki and received approval from the ethics committee of China Medical University Hospital. Informed consent for lens fitting and data use was obtained from each patient’s guardian as part of routine clinical care.

### Participants and eligibility

Children aged 6–18 years with documented myopia (SE ≤ −0.75 D) who were newly prescribed MiSight 1 Day lenses and had at least 12 months of follow-up were eligible. Patients with ocular pathology, systemic diseases that affect refraction, or incomplete biometric data were excluded. Systemic diseases affecting refraction included diabetes, connective tissue disorders, and chronic steroid use. If both eyes met eligibility, one eye was randomly selected, typically the right eye, to maintain independence. For patients switching from orthokeratology, a minimum washout period of 4 weeks was required before baseline measurements.

### Lens fitting and treatment exposure

MiSight 1 Day lenses (omafilcon A; CooperVision) are daily disposable soft hydrogel lenses with a central correction zone and concentric dual-focus rings (+2.00 D) designed to provide clear central vision and peripheral myopic defocus. Lenses were fitted by licensed optometrists or ophthalmologists following manufacturer guidelines. Monotherapy was defined as MiSight wear without concurrent atropine or other myopia control optics; adjunctive therapy referred to MiSight combined with pharmacologic or optical co-treatment. Adherence was assessed from clinician notes; cases with documented persistent noncompliance leading to discontinuation of MiSight were excluded from the final analysis.

### Measurements

Baseline and follow-up SE and AL were extracted from clinical records. SE was measured by non-cycloplegic autorefraction in most cases (70%), and by cycloplegic refraction in the remainder (30%). Cycloplegic refraction used 1% cyclopentolate when clinically indicated, and analyses were adjusted for cycloplegia status. AL was measured using non-contact optical biometry (for example, IOLMaster or Lenstar). Outcomes were annualized for analysis. Annualization used the change in value divided by the exact follow-up interval in years. Follow-up intervals between 10 and 18 months were permitted in the primary analysis.

### Visual acuity and safety

Visual acuity at baseline and follow-up was recorded using Snellen charts. A threshold of 20/25 or better was prespecified as satisfactory functional vision. For summary statistics, Snellen values were converted to logMAR. Adverse events were identified through retrospective review of routine clinical records, focusing on major contact lens related complications and documented discontinuation.

### Outcomes

The primary outcome was annualized axial elongation (mm/year). The secondary outcome was SE progression (D/year). Subgroup analyses were prespecified for age, baseline AL percentile, and prior treatment.

### Normative reference and suppression metric

To contextualize growth, each child’s AL change was compared with Taiwanese age-, sex-, and baseline AL percentile–matched reference data (*N* = 1,623) reported by the National Taiwan University Hospital group (5). Expected annual AL growth from the matched percentile was subtracted from observed growth to derive an axial elongation suppression estimate for each child. A suppression of ≥ 0.10 mm/year was considered clinically meaningful. Effectiveness was also cross-referenced with MiSight clinical trials, including the 3-year randomized trial by Chamberlain et al. (3) and Spanish MASS studies (4).

### Statistical analysis

All analyses were performed in R version 4.2.2. Two-sided *p* values < 0.05 were considered statistically significant. Annual changes in SE and AL were summarized as mean ± SD. Differences across age groups and treatment histories were evaluated using ANOVA or Kruskal–Wallis tests, as appropriate. Axial elongation suppression was calculated as the difference between observed annual AL change and the expected change derived from percentile matching. For subgroup analyses, pairwise comparisons were performed between each subgroup and the prespecified reference category, with *p* values adjusted using the Holm method.

## Results

### Cohort characteristics

A total of 368 eyes from 368 pediatric patients (mean age 11.8 ± 2.3 years; 52% female) were shown in Table 1. Among the cohort, 321/368 had ≥12 months of follow-up (87%), and 20% had ≥24 months. Baseline mean spherical equivalent (SE) was −3.57 ± 1.83 D and axial length (AL) was 24.93 ± 1.04 mm. At baseline, 24% had SE ≤ −5.00 D and 16% had AL ≥ 26.0 mm. The average follow-up duration was 19.2 ± 5.0 months.

**Table 1:** Baseline characteristics of the MiSight cohort at initiation.

Variable	Value
Number of eyes	368
Mean age (years)	11.8 ± 2.3
Female (%)	52%
Baseline SE (D)	−3.57 ± 1.83
Baseline AL (mm)	24.93 ± 1.04
Cycloplegic refraction (%)	30%
Non-cycloplegic refraction (%)	70%

Data presentation: mean ± SD or *n* (%), as appropriate. SE, spherical equivalent; AL, axial length.

### Primary and secondary outcomes

The mean annual SE progression was −0.41 ± 0.42 D/year, with subgroup analysis showing slower progression among children aged 8–11 compared with older adolescents. The mean annual AL elongation was 0.17 mm/year, which was significantly below the expected growth based on normative Taiwanese data (mean matched percentile-adjusted growth: 0.23 mm/year), indicating a mean suppression of 0.06 mm/year. This corresponds to a relative reduction of ~27% versus expected growth (0.06/0.23). The data were demonstrated in Table 2.

**Table 2:** Annualized refractive and axial outcomes with percentile-matched suppression.

Parameter	Value
SE Progression (D/year)	−0.41 ± 0.42
AL Elongation (mm/year)	0.17 ± 0.17
AL Suppression (vs. Taiwanese Percentile, mm/year)	0.06 ± 0.16
AL suppression ≥ 0.10 mm/year	43.2%
20/25 VA or Better (%)	98%

Data presentation: mean ± SD (95% CI) where applicable. SE, spherical equivalent; AL, axial length.

Interindividual variability was observed in percentile-matched AL suppression. Among eyes with available matched expected growth (*n* = 317), 137 eyes (43.2%) achieved AL suppression ≥ 0.10 mm/year. Median AL suppression was 0.071 mm/year.

### Refraction and lens prescription

Vision outcomes were favorable, with 98% of eyes achieving ≥20/25 visual acuity on MiSight lenses. The initial MiSight prescription averaged −3.25 ± 1.51 D. Non-cycloplegic refraction accounted for 70% of measurements (~258 eyes), while ~30% underwent cycloplegic refraction. Refractive change trends were consistent with expected treatment effects in both groups.

### Prior treatment subgroups

Subgroup analyses are summarized in Table 3. Compared with children with no prior treatment or unknown history (*n* = 201), those with prior atropine use (*n* = 107) showed lower annual AL elongation (0.140 ± 0.159 vs. 0.178 ± 0.147 mm/year, *p* = 0.024) and a numerically higher AL suppression (0.096 ± 0.162 vs. 0.056 ± 0.145 mm/year, *p* = 0.113). Children with prior Ortho-K use (*n* = 48) did not differ significantly from the reference group in annual AL elongation (0.180 ± 0.222 mm/year, *p* = 0.191) or AL suppression (0.015 ± 0.198 mm/year, *p* = 0.295).

**Table 3:** Stratified annual axial outcomes in MiSight-treated children.

Subgroup	*n*	AL Elongation (mm/year)	*p* value	AL suppression (mm/year)	*p* value
Stratified by prior treatment history					
No prior treatment	201	0.178 ± 0.147	Reference	0.056 ± 0.145	Reference
Prior Atropine	107	0.140 ± 0.159	0.024*	0.096 ± 0.162	0.113
Prior Ortho-K	48	0.180 ± 0.222	0.191	0.015 ± 0.198	0.295
Stratified by age group					
6–9	64	0.235 ± 0.155	Reference	0.113 ± 0.158	Reference
10–12	148	0.201 ± 0.178	0.072	0.040 ± 0.182	0.025*
13–18	144	0.102 ± 0.123	<0.001*	0.061 ± 0.134	0.033*
Stratified by baseline AL percentile group					
<50th	54	0.136 ± 0.159	Reference	0.024 ± 0.157	Reference
50-74th	95	0.153 ± 0.142	0.238	0.064 ± 0.140	0.330
> = 75th	207	0.182 ± 0.172	0.045*	0.071 ± 0.170	0.273

*: *p*<0.05. Data presentation: mean ± SD (and 95% CI if space permits).

AL suppression was defined as observed annual AL elongation minus expected annual AL growth derived from age-, sex-, and baseline percentile-matched Taiwanese normative data.

*p* values are pairwise comparisons versus the reference group with Holm adjustment. AL indicates axial length; Ortho-K indicates orthokeratology.

**Figure 1:**
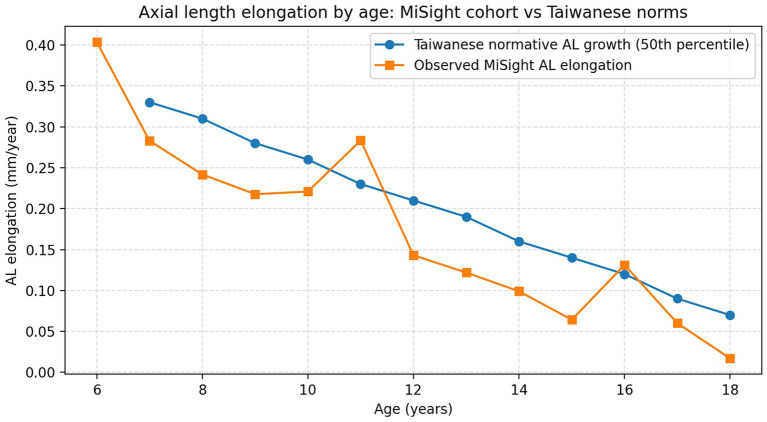
Observed annual AL elongation in MiSight-treated children is plotted by age and compared with the Taiwanese normative AL growth curve (50th percentile) for visual reference. Percentile-matched expected AL growth was used to calculate AL suppression at the individual level.

When stratified by age, annual AL elongation decreased with increasing age. Compared to children aged 6–9 years (*n* = 64; 0.235 ± 0.155 mm/year), AL elongation was 0.201 ± 0.178 mm/year in those aged 10–12 years (*n* = 148, *p* = 0.072) and 0.102 ± 0.123 mm/year in those aged 13–18 years (*n* = 144, *p* < 0.001). In percentile-matched analyses, AL suppression was 0.113 ± 0.158 mm/year in the 6–9-year group and remained significant in both the 10–12-year group (0.040 ± 0.182 mm/year, *p* = 0.025) and the 13–18-year group (0.061 ± 0.134 mm/year, *p* = 0.033) (Table 3). Figure 1 illustrates observed annual AL elongation by age in the MiSight cohort compared with Taiwanese normative growth (50th percentile), supporting the percentile-based suppression framework.

When stratified by baseline AL percentile, children with baseline AL ≥ 75th percentile (*n* = 207) showed higher annual AL elongation than those with baseline AL < 50th percentile (*n* = 54) (0.182 ± 0.172 vs. 0.136 ± 0.159 mm/year, *p* = 0.045). However, AL suppression did not differ significantly across percentile subgroups (*p* = 0.330 for 50–74th percentile; *p* = 0.273 for ≥ 75th percentile, each vs. < 50th percentile) (Table 3).

### Safety outcomes

Safety outcomes were assessed based on diagnoses and narrative documentation in routine clinical records. No cases of corneal ulcer were recorded during follow-up. Two patients developed recurrent corneal erosion while wearing MiSight lenses.

## Discussion

This large real-world study supports the clinical utility of MiSight 1 Day for myopia control in Taiwanese children. We observed a mean axial elongation of 0.17 mm/year and a refractive progression of −0.41 D/year over a mean follow-up of 19.2 months. When compared with percentile-matched Taiwanese norms, the mean axial length (AL) suppression was 0.06 mm/year, consistent with efficacy reported in randomized trials (3, 4). This corresponds to an approximate 27% relative reduction versus expected growth based on local norms.

The magnitude of effect suggests that MiSight retains effectiveness outside trial settings. In the 3-year RCT by Chamberlain et al. (3), MiSight reduced axial elongation by 52% and myopic progression by 59% compared with single-vision spectacles. Visual performance was favorable, with 98% achieving 20/25 or better, supporting practicality in clinic workflows. Younger children in our cohort showed slower progression than older adolescents, aligning with the concept that earlier intervention yields greater benefit.

Using percentile-adjusted AL comparisons based on local biometric references is a methodological strength. East Asian children, including those in Taiwan, demonstrate faster axial growth than many Western cohorts (1, 2). Therefore, interpreting myopia control outcomes using Taiwanese age- and sex-specific normative curves improves clinical relevance and reduces bias that may arise when applying non-local growth expectations (5). This approach allows treatment effects to be evaluated relative to the ocular growth trajectory most applicable to the target population.

For comparison with prior studies, we compared our annualized outcomes with established MiSight studies. In our cohort, mean AL elongation was 0.17 mm/year and mean SE progression was −0.41 D/year. In the 3-year randomized trial by Chamberlain et al. (3), annualized AL elongation was approximately 0.30 mm/year in MiSight wearers versus 0.62 mm/year in controls, with substantial reduction in myopic progression in the MiSight group (4). Although differences in follow-up structure and real-world practice conditions may influence absolute annualized values, our results are directionally consistent with these trial benchmarks. Visual performance was also favorable in routine care, with 98% achieving 20/25 or better, supporting feasibility in clinical practice.

Differences between routine practice and trial settings may partly explain variation in absolute annualized values. In our real-world cohort, adherence was not objectively measured, environmental factors such as outdoor time and near work were not controlled, and baseline characteristics were more heterogeneous, including a wider age range and diverse prior myopia control histories. These features reflect daily practice and may contribute to interindividual variability in treatment response.

Prior treatments were common in our cohort. Approximately one quarter had previously used atropine and a smaller proportion had worn orthokeratology before initiating MiSight. In subgroup analyses, children with prior atropine use showed lower annual AL elongation than the reference group, while AL suppression was numerically higher but did not reach statistical significance. This finding is compatible with prior reports suggesting that sequential or combination strategies involving atropine can remain effective when transitioning to or adding optical myopia control interventions (6). In contrast, the prior Ortho-K subgroup showed no significant difference in annual AL elongation or AL suppression compared with the reference group, but variability was greater. These findings suggest that MiSight can be used after prior myopia control therapies in routine practice, although responses may be heterogeneous, particularly after orthokeratology. Prospective studies with standardized washout and scheduled measurements would help clarify treatment transitions and the extent of any rebound effect.

Axial length is an appropriate structural endpoint for myopia control. SE can be influenced by accommodation and optical corrections, whereas AL tracks ocular growth (7–9). AL suppression was defined as observed annual AL elongation minus the percentile-matched expected growth derived from Taiwanese age-, sex-, and baseline percentile-specific references, and was therefore calculated in eyes with available matched expected values. This provides an individualized estimate of axial slowing relative to the ocular growth trajectory most relevant to Taiwanese children. We interpreted this magnitude alongside response variability, rather than relying on a single mean value alone. Prior literature underscores AL’s relevance to downstream risks such as myopic maculopathy (8). Our data therefore support the view that MiSight may reduce long-term morbidity by moderating structural elongation. In settings with constrained resources, prioritizing AL monitoring alongside SE can improve treatment decisions and follow-up efficiency.

The study confirms that MiSight can maintain high visual performance, with 98% of children achieving 20/25 vision or better. Ethnic and regional considerations are essential in evaluating myopia control interventions. Taiwanese children, as shown in previous NTUH studies, exhibit faster axial elongation than European peers (5). Our comparison with international axial growth data, including German and Chinese cohorts, underscores ethnic disparities in myopia development and supports the use of localized biometric references for treatment evaluation (10, 11). For example, the 50th percentile axial length change in Taiwanese children was higher than in German counterparts by approximately 0.11 mm/year, a differential nearly equivalent to MiSight’s suppression effect. The effect of prior myopia control treatment, such as atropine or orthokeratology, appears limited in influencing subsequent MiSight efficacy, although a small Ortho-K subgroup showed slightly greater AL growth. This could reflect rebound effects or measurement bias (12, 13).

This study’s strengths include a large, multicenter cohort and the use of percentile-matched axial length comparisons, which improves clinical interpretability and aligns evaluation with local growth patterns. The findings were comparable to controlled trial benchmarks and appeared adaptable to diverse clinical workflows in Taiwan.

Limitations arise from the retrospective, real-world design. First, this study did not include a parallel untreated or single-vision control group, and treatment effects were interpreted using Taiwanese normative references rather than a contemporaneous control cohort. Refraction was not uniformly cycloplegic, with 70% measured by non-cycloplegic autorefraction, which may introduce variability (14). Treatment adherence, including daily wear duration and replacement schedule, was not objectively recorded, limiting adherence analyses (7, 15–17). Because adherence was assessed only through routine follow-up and caregiver reporting, differential adherence may have introduced adherence-related bias. Follow-up intervals and refitting protocols varied across clinics, which may affect longitudinal consistency. Environmental and familial factors such as time outdoors, near work, and parental myopia were unavailable for adjustment and are known modifiers of progression (18–21). Minor adverse events were not systematically captured in the available records, and safety assessment therefore primarily reflected major events and clinically documented diagnoses. Device-level differences in biometry and site-level practice variation may have introduced small, unmeasured effects. Despite these constraints, the multicenter setting reflects routine practice and supports external validity.

## Conclusion

MiSight 1 Day lenses provide meaningful myopia control in Taiwanese children in routine care. Axial length growth was suppressed by 27% relative to age-, sex-, and percentile-matched Taiwanese norms, and effects were consistent across prior treatment groups. These findings support the translational effectiveness of MiSight and underscore the value of regional normative benchmarks for clinical interpretation.

## Data Availability

The raw data supporting the conclusions of this article will be made available by the authors, without undue reservation.
